# The development and evaluation of the worker-occupation fit inventory

**DOI:** 10.1186/s12889-023-17080-x

**Published:** 2023-11-06

**Authors:** Keyao Lv, Ruican Sun, Xiaofang Chen, Yajia Lan

**Affiliations:** 1 Mianyang Center for Disease Control and Prevention, Mianyang, 621000 People’s Republic of China; 2 Department of Preventive Medicine, School of Public Health, Chengdu Medical College, Chengdu, 610500 People’s Republic of China; 3 Department of Environmental Health and Occupational Medicine, West China School of Public Health and West China Fourth Hospital, Sichuan University, No. 16, Section 3, Renmin South Road, Chengdu, 610041 China

**Keywords:** Occupational stress, Worker-occupation fit, Item response theory

## Abstract

**Background:**

Person-environment fit (PEF) theory, one of the foundational theories of occupational stress, has primarily found applications in organizational behavior and human resource management. Given the alignment between the definition of occupational stress and the essence of PEF, we introduced the concept of worker-occupation fit (WOF). To validate our theoretical model, the development of an instrument to measure WOF becomes imperative.

**Methods:**

The Worker-Occupation Fit Inventory (WOFI) comprises three dimensions: personal trait fit (PTF), need-supply fit (NSF) and demand-ability fit (DAF). Job-related mental disorders (JRMDs) were assessed using the DASS-21. During the pre-investigation, items of the WOFI underwent screening through classic test theory (CTT) analysis. In the formal investigation, item response theory (IRT) analysis was employed to evaluate the selected items. The relationship between WOF and JRMD was verified by Pearson’s correlation analysis and multiple logistic regression analysis.

**Results:**

The initial version consisted of 26 items. Three common factors were extracted by exploratory factor analysis (EFA): 6 items were included in the PTF, 6 items were included in the NSF, 4 items were included in the DAF, and 10 items were deleted because of unacceptable factor loadings. The confirmatory factor analysis (CFA) verified the structure of the WOFI with χ^2^/df = 1.822, CFI = 0.947, and SRMSR = 0.056. The Cronbach's alpha coefficients of the PTF, NSF, and DAF were 0.91, 0.92, and 0.80, respectively. In IRT analysis, the discrimination values of all items ranged from 1.25 to 2.53, and the difficulty values of all items ranged from -6.28 to 1.30 (with no difficulty of reversal). The WOF was negatively related to job-related stress (*r* = -0.34, *p*<0.001), anxiety (*r* = -0.37, *p*<0.001), and depression (*r* = -0.41, *p*<0.001). The multiple logistic regression analysis suggested that a high level of WOF was a protective factor against job-related mental disorders, with *OR*s all less than 1 (*p*<0.001), and a low level of WOF was a risk factor for job-related mental disorders, with *OR*s all more than 1.0 (*p*<0.001).

**Conclusions:**

The results of CTT and IRT analysis indicated that the WOFI exhibits reliability and validation. The WOF effectively predicted job-related mental disorders. Subsequent studies will delve into the influence of WOFI on diverse professions and various health outcomes.

**Supplementary Information:**

The online version contains supplementary material available at 10.1186/s12889-023-17080-x.

## Background

As industrialization and urbanization have advanced significantly, the work environment is undergoing profound changes, and the fit level between workers and their work environment has become emerged as a novel stressor. Worker-occupation fit (WOF) is defined as the fit level between workers’ personal characteristics, needs and abilities, and the cultural atmosphere, supplies and demands within the work environment [1]. The concept of WOF originates from person-environment fit theory (PEF theory), initially proposed by Parsons in his 1909 work, “*Choosing a Vocation*” [2]. PEF stands as a fundamental concept within the domains of organizational behavior and human resource management [3–5]. While a substantial body of research has primarily focused on exploring the impacts of PEF on workers’ performance, turnover and organizational performance, relatively little attention has been given to the relationship between fit and occupational stress. The National Institute for Occupational Safety and Health (NIOSH) defined occupational stress as “the harmful physical and emotional responses that occur when the requirements of the job do not match the capabilities, resources, or needs of the worker” in *Stress at Work*. Given the consistency between this definition of occupational stress and the essence of WOF, we have formulated a theoretical model delineating the health effects of WOF on occupational stress. According to this model, we posit that workers’ health benefits is positively influenced by a high level of WOF, whereas misfit may induce occupational stress, thereby jeopardizing workers' well-being.

Person-environment fit theory, one of the earliest theories concerning occupational stress [6], has yet to reach its full potential in applied research due to a lack of adequate measurement tools. The measurement of WOF can be approached in two ways: perceived fit and objective fit. Existing studies overwhelmingly demonstrate that perceived fit exhibits superior predictive abilities for target outcomes [7]. In 2016, Chunag et al. established the Perceived Person-Environment Fit Scale (PPEFS) from the perspective of organizational psychology [8]. Their study highlighted the significant predictive power of perceived person-environment fit concerning role behavior, job satisfaction, turnover, and organizational citizenship behavior. Recognizing the substantial impact of workers' perceived fit on occupational stress and job-related diseases, our research is specifically designed to concentrate on workers' perceived fit. Drawing upon the knowledge base of occupational stress and building upon prior research by Cable and DeRue [7], our objective is to build the Worker-Occupation Fit Inventory (WOFI). The dimensions of the WOFI have been formulated as follows: personal traits fit, need-supply fit, and demand-ability fit.

To establish a reliable and valid measurement for WOF, both classic test theory (CTT) analysis and item response theory (IRT) analysis will be employed to assess the scale. CTT analysis, a conventional method in scale development, has limitations such as reliance on sample parameters and discrepancies in scale difficulty and participant ability, leading to less accurate reliability estimation. IRT compensates for these shortcomings well. IRT effectively addresses these shortcomings by offering detailed insights into each item's performance, unaffected by sample variations [9]. Petrillo, in their evaluation of patient-reported outcome measures [10], found that while the results from both methods were similar, IRT provided more intricate information. The same conclusion was drawn in a study that simplified and evaluated the Toronto Empathy Questionnaire [11].

Accurate identification, measurement and evaluation of mental disorders within occupational populations form a crucial basis for implementing tailored interventions to enhance mental health in the workplace. The purpose of our study is to develop the WOFI, which is supposed to meet the following criteria: has great consistency with the theoretical framework and structure, performs satisfactorily in measuring WOF, and include items suitable for JRMD screening and monitoring in occupational populations.

## Methods

### Procedures and participants

**Fig. 1:**
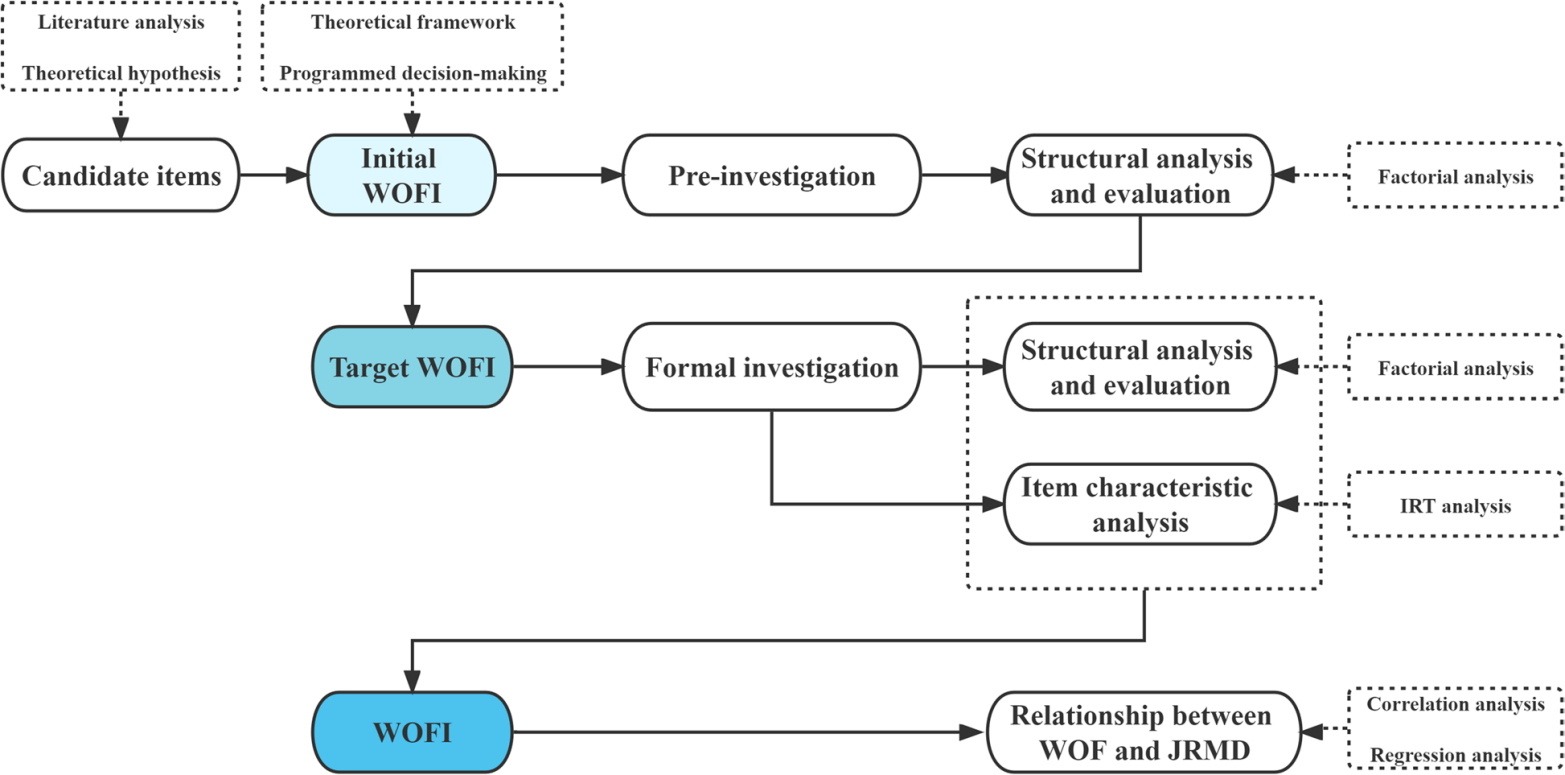
The flowchart

This research is consist of a pre-investigation and a formal investigation. Prior to the investigations, the candidate items were selected through literature analysis and theoretical hypotheses. The initial WOFI was established based on the theoretical framework and programmed decision-making process. The WOFI was refined through both a pre-investigation and a formal investigation. It was then utilized to analyze the relationship between WOF and JRMD. The research process is illustrated in Fig. 1.

The enrollment of participants in both stages followed the same inclusion and exclusion criteria. The inclusion criteria were as follows: (1) individuals employed in the hospital; (2) individuals with a minimum work experience of 1 year; and (3) individuals who voluntarily participated in this survey and provided informed consent. The exclusion criteria were as follows: (1) participants on leave during this survey (including sick leave, maternity leave, personal leave and those who pursuing further study); (2) participants with incomplete or ineligible questionnaires (< 80% completed); and (3) individuals currently diagnosed with tumors, severe organic diseases, or mental disorders.

In the pre-investigation, the sample size was calculated as 108 (more than 9% of the formal investigation sample size). The participants were recruited among medical workers who participated in physical examinations in a hospital in Sichuan Province. A total of 128 questionnaires were collected in the pre-investigation, and a total of 8 participants with less than 1 work year or invalid questionnaires were excluded. The response rate was 93.8%, and the sample size was in line with expectations (more than 108). The average age of the participants in the pre-investigation was 32.7 (SD = 7.9). A total of 38.3% (*n* = 46) of them were male, 61.7% (*n* = 74) were female. In terms of occupation, 42.5% (*n* = 51) of them were physicians, 28.3% (*n* = 34) were nurse, and 28.3% (*n* = 35) were hospital administrators. The pre-investigation was conducted to screen items for the target WOFI.

In the formal investigation, the target WOFI was performed. Following Lachin's method [12], we calculated the sample size parameters based on preliminary research findings. With a correlation coefficient of -0.20 under the null hypothesis and a correlation coefficient limit of -0.30, the sample size was computed as 925 cases. Setting a design coefficient of 0.20, the final sample size after correction is 1200 cases.

Participants were recruited from medical workers in a hospital located in Sichuan Province and another in Henan Province. A total of 1,183 questionnaires were distributed during the formal investigation (562 questionnaires in Sichuan province, 621 questionnaires in Henan province). Three participants on sick leave were excluded. Additionally, ten questionnaires from participants with less than 1 year of work experience and eight duplicate or invalid questionnaires were excluded. Ultimately, 1,162 valid questionnaires were collected, resulting in a response rate of 98.2%. Of the participants, 24.7% were male (*n* = 287), and 75.3% were female (*n* = 875). The occupational composition was as follows: 25.3% (*n* = 294) were physicians, 55.3% (*n* = 643) were nurses, 6.6% (*n* = 76) were medical technologists, 4.1% (*n* = 48) were pharmacists, and 8.7% (*n* = 101) were hospital administrators (see other characteristics in Table 1).

**Table 1:** Demographic and occupational characteristics of the participants in the formal study

Variable	Groups	N	%
Sex	Male	287	24.7
	Female	875	75.3
Age (year)	20 ~	341	29.4
	30 ~	449	38.6
	40 ~	372	32.0
Marital status	Single	253	21.8
	Unmarried cohabitation	17	1.5
	Married	851	73.2
	Divorced	41	3.5
Education	College	243	20.9
	Bachelor	813	70.0
	Master or above	106	9.1
Income (yuan per month)	< 3,000	137	11.8
	3,000 ~	559	48.1
	5,000 ~	430	37.0
	10,000 ~	36	3.1
Work experience (year)	1 ~	259	22.3
	5 ~	262	22.5
	10 ~	337	29.0
	20 ~	304	26.2
Work hours (per week)	< 40	210	18.1
	40 ~	475	40.9
	50 ~	266	22.9
	60 ~	211	18.1
Occupational categories	Physician	294	25.3
	Nurse	643	55.3
	Medical technologist	76	6.6
	Pharmacist	48	4.1
	Hospital administrator	101	8.7
Job title	Senior	234	20.1
	Intermediate	383	33.0
	Junior	482	41.5
	No title	63	5.4
Night shift (per week)	0	387	33.3
	1 ~	560	48.2
	3 ~	182	15.7
	5 ~	33	2.8
Exercise (per week)	0	375	32.2
	1 ~	556	47.9
	3 ~	127	10.9
	5 ~	104	9.0
Drink	Never	784	67.5
	Rarely	336	28.9
	Often	42	3.6
Smoke	Never	1,029	88.5
	Rarely	53	4.5
	Often	80	6.9
Traumatic events	YES	133	11.5
	NO	1,029	88.5

### Measures

#### Worker-occupation fit inventory

Following the theoretical model, we established the Worker-Occupation Fit Inventory (WOFI) based on the perspective of perceived fit. The dimensions of WOFI include personal traits fit, need-supply fit and demand-ability fit. The personal traits fit dimension originates from supplementary fit, which occurs when an individual and an organization possess similar or matching characteristics [13]. In this context, personal trait fit was defined as the fit level between workers’ personality traits and organizational values. Personality is a stable but complex pattern of thoughts, feelings, and behaviors of individuals [3]. Consequently, we meticulously gathered personality traits known to influence occupational stress through extensive literature review, forming an item pool for personal traits fit. Need-supply fit and demand-ability fit are mainly connected with job characteristic. Hackman and Oldham’s job characteristic model (JCM) posits that core job characteristic significantly impact workers’ key psychological state [14]. Guided by this perspective, we collected questionnaires, including Hackman’s Job Diagnostic Survey [15], Karasek’s Job Content Questionnaire [16], Smis’s Job Characteristic Inventory [17], and Morgeson and Humphrey’s Work Design Questionnaire [18]. Items were chosen from those questionnaires, constituting the item pools for need-supply fit and demand-ability fit. All selected items were compiled into an EXCEL spreadsheet to form the item pool for the WOFI.

**Fig. 2:**
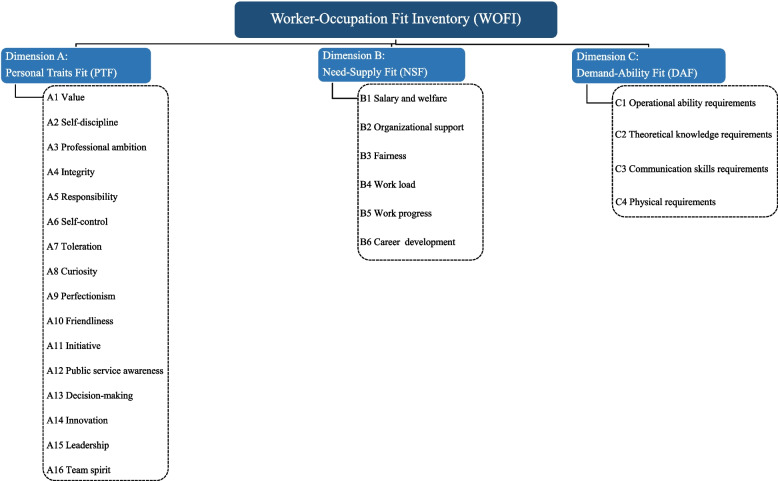
The framework of the WOFI

Items were preliminarily screened by a decision-making panel utilizing a programmed decision-making approach. Through extensive literature review, analysis, and reference to existing scales, the decision-making panel selected items aligning with the concepts of each dimension. In the personal traits fit dimension, 16 items such as values, self-discipline, and self-motivation were included, numbered from A1 to A16. In the demand–supply fit dimension, 6 items such as salary and welfare satisfaction, organizational support, and fairness were incorporated, numbered from B1 to B6. The demand-ability fit dimension comprised 4 items including operational ability requirements and theoretical knowledge requirements, numbered from C1 to C4. The framework of the WOFI is shown in Fig. 2. To ensure cultural relevance and participant comprehension, the decision-making panel adapted the selected items to fit the Chinese cultural context. Participants rated these items on a five-point Likert scale, ranging from 0 (“Strongly Misfit”) to 4 (“Totally Fit”).

#### Depression anxiety stress scale

Depression Anxiety Stress Scale (DASS-21), developed by Lovibond P.F and Lovibond S.H [19], has been translated into various languages and used widely. In a study involving Chinese medicine workers [20], the DASS-21 demonstrated high reliability, with a Cronbach’s alpha coefficient exceeding 0.85 (0.95). This scale comprises three subscales assessing stress, anxiety and depression, each consisting of seven items. The DASS-21 was scored on a four-point Likert scale, ranging from 0 (“Strongly Disagree”) to 3 (“Totally Agree”). To calculate the total scores for each subscale, the sum of the scores for the seven items was doubled. Higher scores indicated more severe mental disorders. A score exceeding 14 indicated stress, score exceeding 7 indicated anxiety, and score exceeding 9 indicated depression [19]. The DASS-21 was employed to evaluate job-related mental disorders, including job-related stress (JRS), job-related anxiety (JRA) and job-related depression (JRD). To ensure accuracy, participants were prompted to recall work-related situations while completing the DASS-21.

### Statistical analysis

#### Classic test theory analysis

CTT analysis was conducted in the pre-investigation. First, item analysis was conducted to evaluate the discriminant validity for each item by comparing the difference between the low group (bottom 27% in score) and the high group (top 27% in score). Items with a *p* value < 0.05 w considered to have good discriminant validity. The Kaiser‒Meyer‒Olkin (KMO) test and Bartlett's test of sphericity were performed to verify that the data were suitable for principal component analysis, and KMO values greater than 0.80 and the *p* values < 0.05 were acceptable. Then, exploratory factor analysis (EFA) was conducted to screen items. The Cattell scree test was used to determine the number of common factors. The Cattell scree test was used to determine the number of common factors. Common factors were extracted by principal component analysis and varimax rotation, and items were filtered and aggregated according to the factor loading of each item. After item screening, confirmatory factor analysis (CFA) was conducted to estimate the factorial structure of the WOFI. For this purpose, three models were evaluated: model 1 was a one-factor-model, and was considered a basal comparison model; model 2 was a two-factor-model, and this model was built to see if there are two dimensions that should be combined; and model 3 was a three-factor-model, and this model was built according to the three dimensions of the WOFI. The goodness-of-fit indices were χ^2^, comparative fit index (CFI), and standardized root mean squared residual (SRMSR). Values equal to or below 3.0 on χ^2^/df, values equal to or above 0.90 on the CFI and values below 0.08 on the SRMR indicated a good model fit. The internal consistency of the WOFI was estimated with Cronbach's alpha coefficient.

#### Item response theory analysis

IRT analysis was conducted in the formal investigation. Cronbach's alpha coefficients were calculated separately for each of the three subscales, with an acceptable range of alpha of more than 0.8. Unidimensionality is one of the basic hypotheses of IRT, which needs to be verified before parameter estimation. The unidimensionality of the three subscales was tested by EFA, and a ratio of the first to second eigenvalue greater than 3.0 was acceptable. The graded response model (GRM) was adapted to perform IRT analysis. Discrimination (a) and difficulty (b) were two important parameters. The discrimination reflected how well the scale distinguishes the participants’ latent. A higher a value indicated better discrimination, a value less than 0.50 indicated insufficient information, and a value higher than 4.0 indicated limited accuracy. Therefore, the value was set in the range of 0.50 to 4.0 [21]. The b values (also called threshold indices) indicated the difficulty of an item. The larger the b value is, the more difficult the items are. When the b values varied from -4 to 4, the difficulty of the item was moderate. In addition, the item characteristic curve (ICC) was used to visualize the parameters. The item information curve (IIC) for each item and the test information curve (TIC) for the WOFI were plotted to show the information amount that each item and the whole inventory provided, with an acceptable range of information amount of more than 1.0.

#### Relationship between WOF and job-related mental disorders

The overall scores of the WOFI were 64, and the WOF level was graded according to the first and third quartiles, with low WOF level scoring no more than 38 points, medium WOF level scoring 39–51 points, and high WOF level scoring 51–64 points. Job-related mental disorders were set as binary outcomes (with or without JRMD) according to scores on the DASS-21. Scores greater than 14, 7, and 9 on the JRS, JRA, and JRD, respectively, indicated one of the job-related mental disorders. To explain the relationship between WOF and JRMDs, three risk models were constructed for JRS, JRA, and JRD. WOF and other variables with statistical significance in Pearson’s correlation analysis were included in the multiple logistic regression model. The incidence risk of JRS, JRA, and JRD was expressed as odds ratios (*OR*s) and 95% confidence intervals (95% *CI*s). A two-tailed *p* value < 0.05 was considered statistically significant.

All data analyses were performed by R software.

## Results

### Classic test theory analysis

The WOFI scores were sorted from high to low, and the top 27% (scored more than 58 points) of the total score were the high group, and the bottom 27% (scored less than 47 points) of the total score were the low group. Significant differences were found between the two groups of scores in all items (all *p* values<0.001, two-sample t-test), indicating that all items had good discriminant validity (see Table S1 in the Supplemental materials). The KMO test was 0.91 and the *p* value was less than 0.01 in Bartlett's test, which suggested that the data were adequate for conducting an EFA. Three common factors were extracted according to the Cattell scree test (see the scree plot in Figure S1). Item A1, A4, A5, A6, A7, A10, A11, and A16 were excluded because their principal factor loadings do not correspond to the concept. Item A2 and A12 were excluded due to their low principal factor loadings. Finally, item A3, A8, A9, A13, A14, and A15 were included in FACTOR1 (PTF), item B1, B2, B3, B4, B5, and B6 were included in FACTOR2 (NSF), and item C1, C2, C3, and C4 were included in FACTOR3 (DAF) (see Table 2).

**Table 2:** The exploratory factor analysis and item induction for the WOFI

Item	Factor loading			Targeted WOFI	
	F1	F2	F3	Dimension	Explanation
A4			0.725		Principal loading dose not correspond to the concept, excluded
A5			0.837		the same
A6	0.734		0.791		the same
A7	0.655		0.671		the same
A10			0.678		the same
A11	0.751		0.814		the same
A16			0.726		the same
A2	0.706		0.658		Low principal loading, excluded
A12	0.611				Low principal loading, excluded
A3	0.771			PTF	Principal loading on F1, reserved
A8	0.720		0.706	PTF	Principal loading on F1, reserved
A9	0.800		0.685	PTF	Principal loading on F1, reserved
A13	0.829		0.729	PTF	Principal loading on F1, reserved
A14	0.840		0.603	PTF	Principal loading on F1, reserved
A15	0.751			PTF	Principal loading on F1, reserved
A1		0.729		PTF	Principal loading dose not correspond to the concept, excluded
B1		0.796		NSF	Principal loading on F2, reserved
B2		0.815		NSF	Principal loading on F2, reserved
B3		0.833		NSF	Principal loading on F2, reserved
B4		0.877		NSF	Principal loading on F2, reserved
B5		0.802		NSF	Principal loading on F2, reserved
B6		0.879		NSF	Principal loading on F2, reserved
C1			0.638	DAF	Principal loading on F3, reserved
C2	0.647		0.757	DAF	Principal loading on F3, reserved
C3			0.683	DAF	Principal loading on F3, reserved
C4			0.611	DAF	Principal loading on F3, reserved

**Fig. 3:**
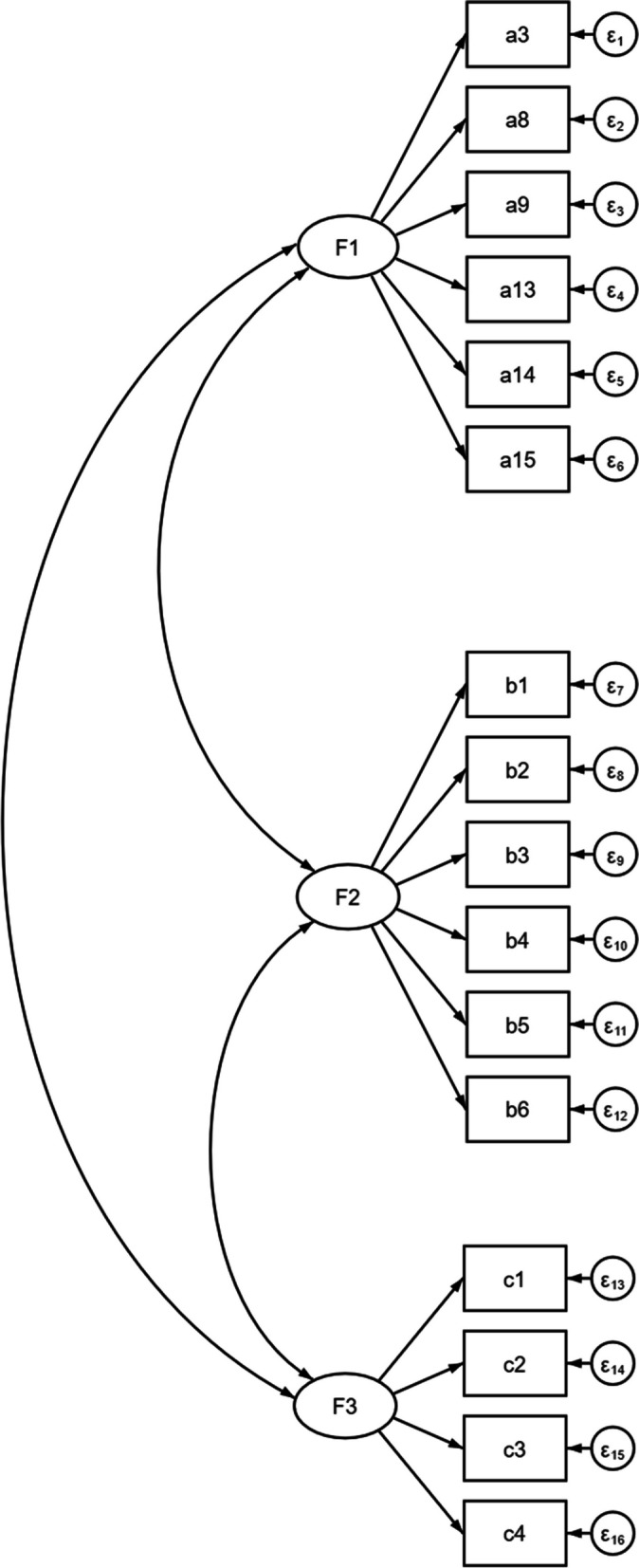
The structure of the three-factor model

Then, CFA was conducted to verify the structure of the WOFI. The results of CFA for the three models are shown in Table 3. Model 3 had the best fit with χ^2^/df = 1.822, CFI = 0.947, and SRMSR = 0.056, and the structure of model 3 is shown in Fig. 3. The results of CFA verified that the WOFI consists of three dimensions, which was consistent with our theoretical assumptions. The Cronbach's alpha coefficient of the WOFI was 0.93 (more than 0.80), indicating great internal consistency of the scale.

**Table 3:** The confirmatory factor analysis for WOFI

Model	χ^2^	df	χ^2^/df	*p*	CFI	SRMSR
Model 3	183.973	101	1.822	< 0.001	0.947	0.056
Model 2	283.895	103	2.756	< 0.001	0.885	0.069
Model 1	671.221	104	6.454	< 0.001	0.640	0.145

### Item response theory analysis

**Fig. 4:**
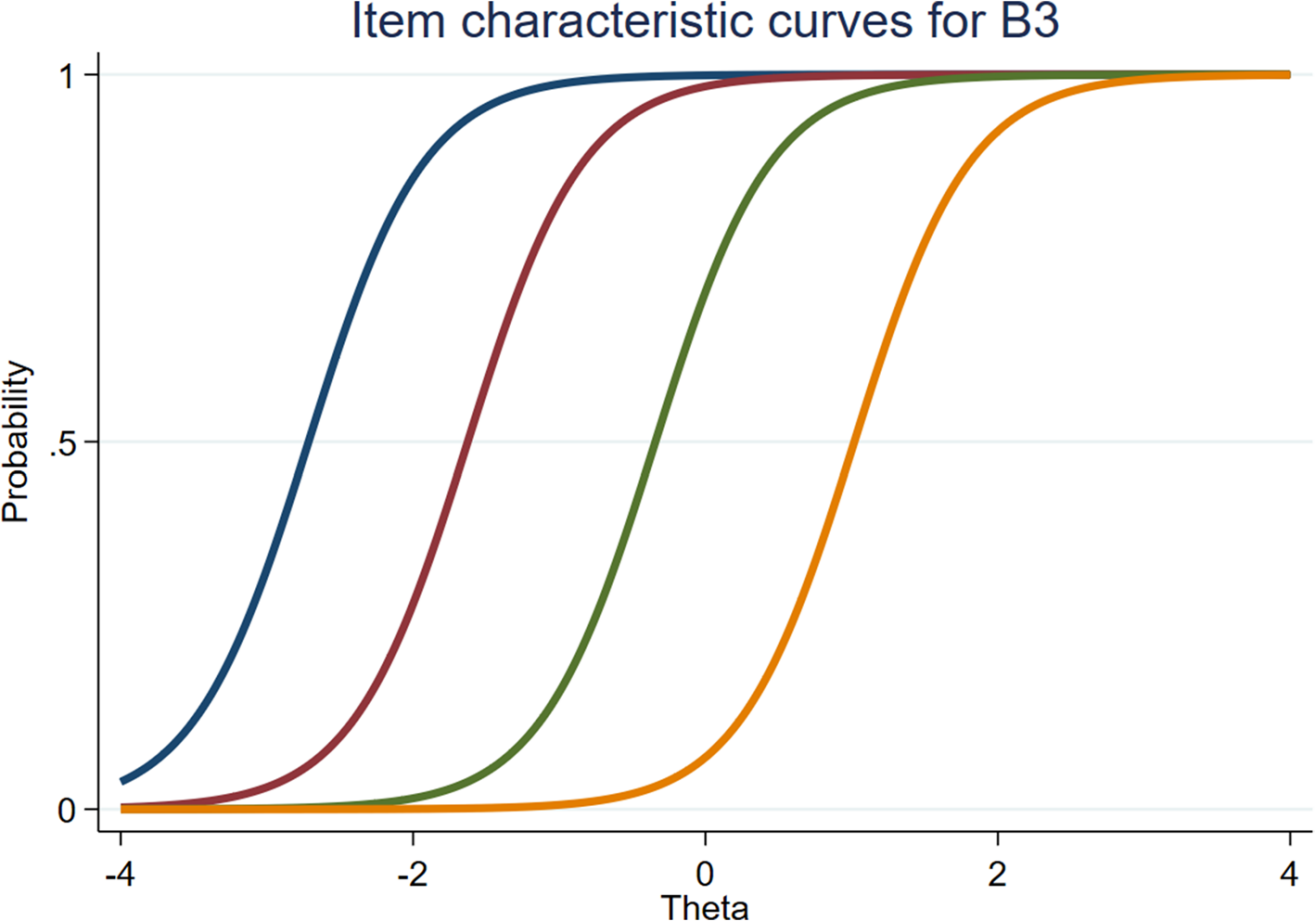
Item characteristic curves for B3

**Fig. 5:**
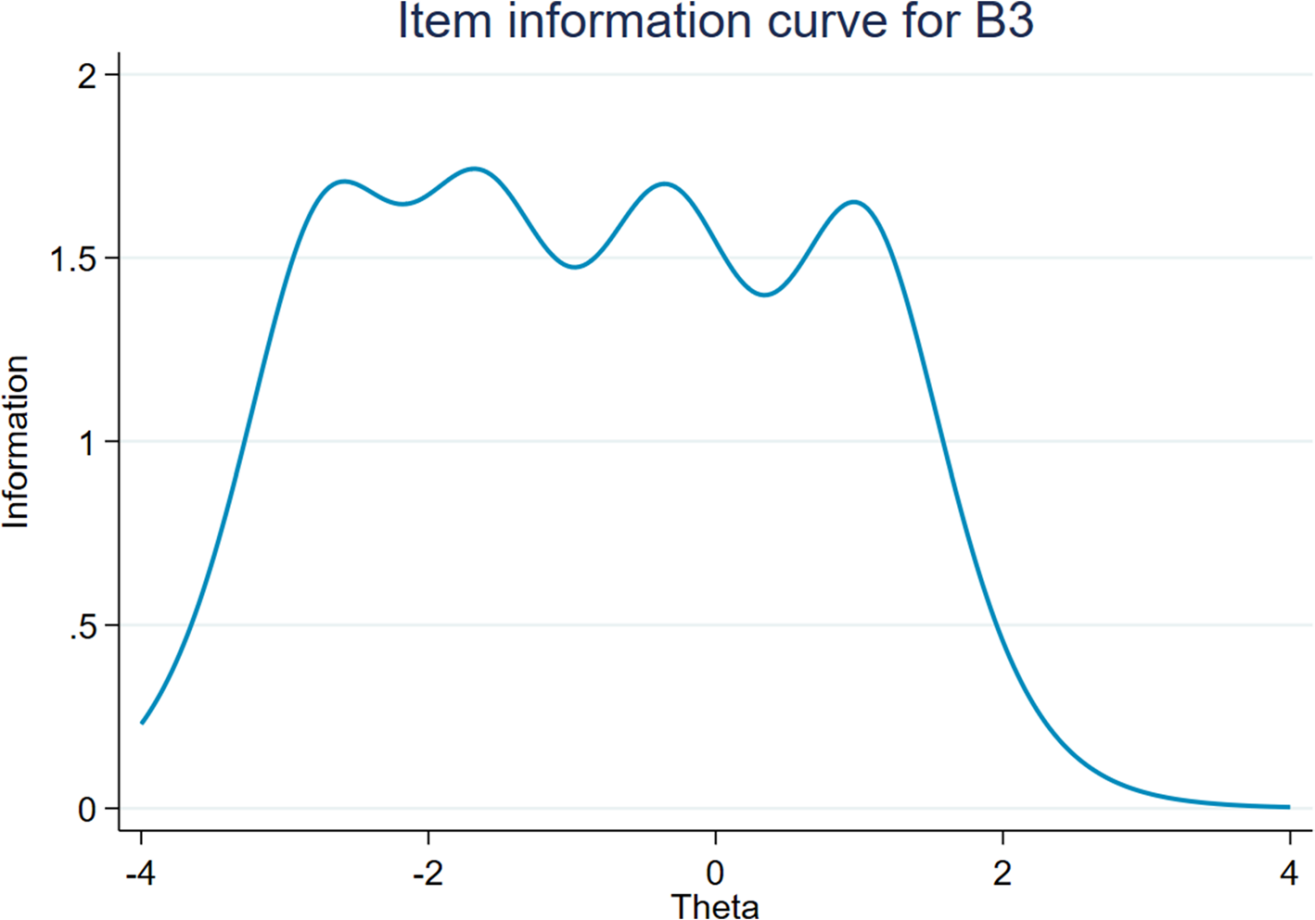
Item information curve for B3

**Fig. 6:**
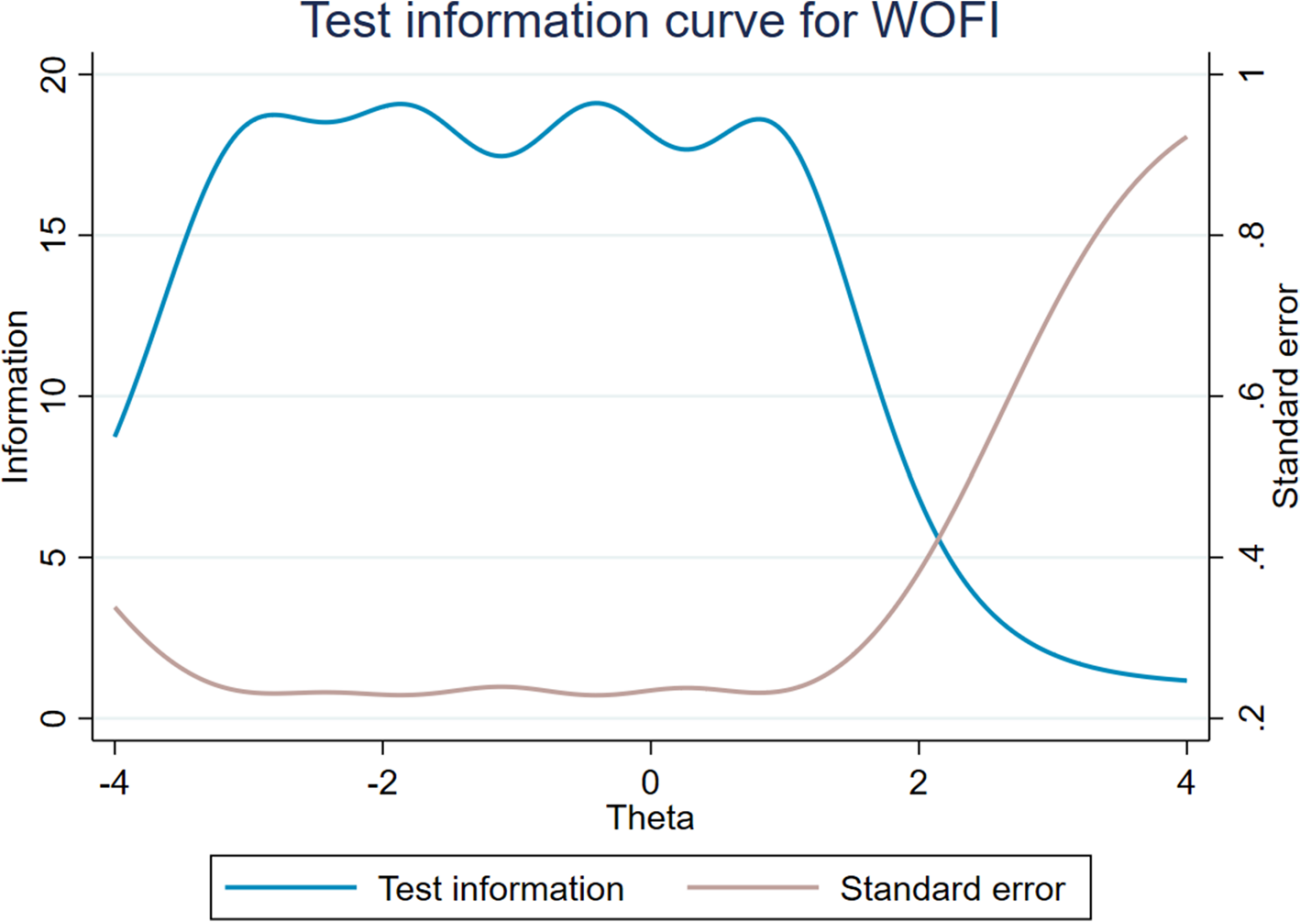
Test information curve for WOFI

In the formal investigation, a large sample was used to conduct IRT analysis. The Cronbach's alpha coefficients of PTF, NSF, and DAF were 0.91, 0.92, and 0.80, respectively. The KMO tests were 0.92, 0.91, and 0.80, and the *p* values were all less than 0.001 in Bartlett's test. The EFA analysis showed that the three subscales met the basic hypothesis of unidimensionality, with values of 189.00, 64.50 and 16.64 (all more than 3) on the ratio of the first to second eigenvalue (see table S2 in Supplemental materials). Table 4 reports the results of IRT analysis for WOFI. The values of all items ranged from 1.25 to 2.53, which were within an acceptable range. The b values of all items ranged from -6.18 to 1.30, and tended to increase monotonically as the difficulty level increased (with no difficulty of reversal). The ICCs, IIC and TIC were used to visualize these results. For all items, each curve of the ICCs plots distinguished different response categories of each option, which was consistent with the results reflected in b values (see Figure S2 in Supplemental materials). IICs for each item showed that each item provided enough information with information amount values all greater than 1.5 (see Figure S3 in Supplemental materials). Figures 4 and 5 show great examples of ICCs and IICs, respectively. The TIC for WOFI indicated that the inventory was also informative (see Fig. 6).

**Table 4:** The results of the IRT analysis for WOFI

Discrimination		Threshold indices				Assessment
	a	b1	b2	b3	b4	
A3	2.17	-3.15	-2.12	-0.76	0.68	Good
A8	2.03	-3.15	-1.89	-0.50	0.87	Good
A9	2.34	-3.15	-1.98	-0.54	0.77	Good
A13	2.30	-3.27	-2.01	-0.59	0.81	Good
A14	2.26	-3.32	-2.00	-0.45	0.84	Good
A15	1.99	-3.29	-2.00	-0.42	0.91	Good
B1	1.91	-2.97	-1.45	0.05	1.30	Good
B2	2.44	-3.08	-1.71	-0.39	0.96	Good
B3	2.53	-2.71	-1.63	-0.34	1.02	Good
B4	2.31	-2.83	-1.69	-0.32	1.10	Good
B5	2.28	-2.77	-1.67	-0.33	1.11	Good
B6	2.34	-2.98	-1.68	-0.26	1.05	Good discrimination, relatively low difficulty
C1	1.36	-5.39	-3.10	-1.06	0.77	Good discrimination, relatively low difficulty
C2	1.32	-5.49	-3.32	-1.08	0.86	Good discrimination, relatively low difficulty
C3	1.25	-6.28	-3.82	-1.23	0.94	Good discrimination, relatively low difficulty
C4	1.47	-5.08	-2.95	-0.92	0.71	Good

### The relationship between WOF and job-related mental disorders

The detection rate of JRS was 48.9% (*n* = 620), the detection rate of JRA was 73.0% (*n* = 935), and the detection rate of JRD was 69.0% (*n* = 874) in the formal investigation. The WOF was negatively related to JRS (*r* = -0.34, *p*<0.001), JRA (*r* = -0.37, *p*<0.001), and JRD (*r* = -0.41, *p*<0.001). Three risk models were constructed with variables including sex, marital status, age, income, occupational categories, job title, work experience, work hours, night shifts, exercise, drinking and traumatic events adjusted. The three models showed that a low level of WOF was a risk factor for JRS(*OR* = 2.15,* p*<0.001), JRA(*OR* = 2.88,* p*<0.001), and JRD(*OR* = 2.57,* p*<0.001), with all *OR*s more than 1.0, and a high level of WOF was a protective factor for JRS(*OR* = 0.15,* p*<0.001), JRA(*OR* = 0.07,* p*<0.001), and JRD(*OR* = 0.09,* p*<0.001) with all *OR*s less than 1.0 (see Table 5).

**Table 5:** Multiple logistic regression analysis of the relationship between WOF and JRMDs

WOF	Job-related stress		Job-related depression		Job-related anxiety	
	*OR* (95%*CI*)	*p*	*OR* (95%*CI*)	*p*	*OR* (95%*CI*)	*p*
Low	2.15(1.57 ~ 2.94)	< 0.001	2.88(1.85 ~ 4.48)	< 0.001	2.57(1.62 ~ 4.07)	< 0.001
Medium	1		1		1	
High	0.15(0.10 ~ 0.22)	< 0.001	0.07(0.05 ~ 0.11)	< 0.001	0.09(0.06 ~ 0.14)	< 0.001

(Variables including sex, marital status, age, income, occupational categories, job title, work experience, work hours, night shifts, exercise, drinking and traumatic events were adjusted in the multiple regression models)

## Discussion

To verify the theoretical model of the worker-occupational-fit occupational-stress health effect, we developed and evaluated the WOFI, and the relationship between WOF and job-related mental disorders was verified. The study was divided into two stages. In the pre-investigation, we accomplished the development of the WOFI and item screening. The initial version of the WOFI consisted of three dimensions with 26 items, included personal trait fit, need-supply fit and demand-ability fit. The items were rated by a five-point Likert scale. This measurement was performed in the pre-investigation, and the items were screened by CTT analysis. After EFA analysis, 6 items were retained in PTF, 6 items were retained in NSF, and 4 items were retained in DAF. The Cronbach's alpha coefficient values for both the WOFI and the three subscales were above 0.8, which suggested a great consistency of the measurement. In the formal investigation, we evaluated the WOFI and verified the relationship between WOF and job-related mental disorders. The evaluation was conducted by IRT analysis, and the discrimination values and difficulty values were within an acceptable range. The multiple logistic regression analysis suggested that a high level of WOF was a protective factor against job-related mental disorders, with all *OR*s less than 1.

A few studies have developed scales for PEF. The most widely used measure was developed by Cable and Derue [7], who measured subjective fit from the perspective of organization psychology. This scale is reliable, validated and focused on the occupational context, which has both theoretical and practical implication for applications of PEF in the field of occupational stress. However, there were some limitations in that we cannot utilize this scale in assessing occupational stress directly. Firstly, the supplementary fit only examined the value congruence between individuals and organizations, but there were many characteristics affecting WOF and workers’ mental health. Secondly, the complementary fit consisted of need-supply fit and demand-ability fit, but this scale only measured the need-supply fit. A multidimensional PEF instrument was developed by Chuang, A. [8], named The Perceived Person-Environment Fit Scale (PPEFS). The PPEFS also measured subjective fit directly and consisted of four subscales, including the Person–Job Fit Scale (PJFS), the Person–Organization Fit Scale (POFS), the Person–Group Fit Scale (PGFS), and the Person–Supervisor Fit Scale (PSFS). The PPEFS considered not only values, but also goals and attitudes, which is in line with the multidimensionality of PEF. The previous scales were most developed as instruments of organizational management, human resource management and employee performance. Research on community psychology [22] also began to adopt PEF theory, and established a scale that met the goals of these studies. Although the previous scales cannot be applied in the area of occupational stress directly, they can be used for reference in our research.

WOFI comprises three dimensions that align with Cable's three-factor conceptual model. The dimensions and item content of WOFI are comprehensive, theoretically addressing the issue of scale universality. The dimension of personal trait fit primarily assesses the fit level between personality traits and organizational values, encompassing 6 items: professional ambition, curiosity, perfectionism, decision-making, innovation and leadership. Kilian's research [23] revealed that a moderate level of professional ambition, combined with the ability to effectively manage occupational stress, may contribute to improved mental health among individuals adhering to masculinity norms. Another study focusing on highly educated young working women emphasized the significant impact of achieving a balance between ambition and the work environment on health outcomes [24]. Regarding perfectionism, a study conducted among athletes demonstrated that both self-oriented and socially prescribed performance perfectionism were correlated with burnout [25]. Curiosity has been identified as a protective factor against anxiety, depression, and suicidal ideation in several studies [26, 27]. Additionally, research has linked decision-making [28], innovation [29] and leadership [30] to the development of various job-related mental disorders. It is essential to recognize that the occupational environment and personality traits mutually influence each other [13]. The contents of need-supply fit and demand-ability fit drawn from widely employed scales assessing job characteristics. These core job characteristics typically encompass skill variety, task identity, task significance, autonomy and feedback. Numerous studies have indicated that job characteristics exert influence on various aspects, including occupational stress [31], smoking behaviors [32] and other health outcomes [33]. In our research, we partitioned these job characteristics into job supply and job demand components to formulate items aligning with the theoretical model.

This study has innovatively developed a work-occupation fit inventory tailored for the professional population based on the Person-Environment Fit theory and the knowledge framework of occupational stress. The WOFI addresses a notable gap in measurement tools, offering substantial contributions to the advancement and application of Person-Environment Fit theory within the realm of occupational health, as well as providing valuable insights into the exploration and clarification of occupational stress mechanisms. Moreover, our research has unearthed the significant role of WOF as a protective factor to cope with occupational stress. Elevating WOF levels has demonstrated the potential to mitigate the risk of occupational stress occurrence. This discovery presents promising avenues for the prevention and intervention of occupational stress.

However, it is important to acknowledge certain limitations in this study. Firstly, the survey was conducted exclusively among healthcare professionals, resulting in a relatively homogeneous study population. In order to diversify the composition of the study subjects, the hospital administrators were also included in the survey. In subsequent research endeavors, it would be beneficial to extend the application and validation of the scale to other professional groups and diverse work settings. Secondly, among the three dimensions of the scale, items related to NSF and DAF dimensions exhibited stronger psychometric properties compared to those for PTF dimension, although they still met the evaluation criteria. Variability in the understanding and discernment of different measurement contents among the participants contributed to performance disparities among the dimensions to some extent. Therefore, ongoing refinement of the theoretical model is necessary to enhance comprehensibility.

### Supplementary Information

**Additional file 1.** Supplemental materials.

## Data Availability

Data may be made available by contacting the corresponding author.

## Abbreviations

WOFI: Work-occupation fit inventory
PEF: Person-environment fit
CTT: Classic test theory
IRT: Item response theory
PTF: Personal traits fit
NSF: Need-supply fit
DAF: Demand-ability fit
DASS-21: Depression, anxiety and stress scale-21
JRMD: Job-related mental disorder
JRS: Job-related stress
JRD: Job-related depression
JRA: Job-related anxiety
EFA: Exploratory factor analysis
CFA: Confirmatory factor analysis
OR: Odds ratio
