# Supplementary material for: The development and evaluation of the worker-occupation fit inventory
Source: BMC Public Health. 2023 Nov 6;23:2163. doi: 10.1186/s12889-023-17080-x (PMC10626709; doi:10.1186/s12889-023-17080-x)
Supplement: Supplementary file 1 — Additional file 1. Supplemental materials. [file 12889_2023_17080_MOESM1_ESM.docx]

Supplemental materials

Table S1 Item analysis for the WOFI（$\bar{x}\pm s$）

| Item | Low group (n = 33) | High group (n = 33) | *t* | *p* |
| --- | --- | --- | --- | --- |
| A1 | 59.89±19.81 | 90.83±7.89 | -8.584 | ﹤0.001 |
| A2 | 64.03±17.60 | 91.20±10.51 | -7.842 | ﹤0.001 |
| A3 | 62.00±15.94 | 90.49±90.71 | -9.027 | ﹤0.001 |
| A4 | 79.77±14.99 | 4.06±0.66 | -6.456 | ﹤0.001 |
| A5 | 76.51±16.699 | 97.77±3.896 | -7.334 | ﹤0.001 |
| A6 | 67.46±17.162 | 94.29±7.094 | -8.547 | ﹤0.001 |
| A7 | 70.03±17.963 | 97.23±4.499 | -8.690 | ﹤0.001 |
| A8 | 61.17±20.052 | 85.49±18.160 | -5.317 | ﹤0.001 |
| A9 | 63.60±21.787 | 91.57±7.739 | -7.157 | ﹤0.001 |
| A10 | 77.89±16.333 | 96.09±7.035 | -6.055 | ﹤0.001 |
| A11 | 67.43±18.735 | 95.66±5.029 | -8.609 | ﹤0.001 |
| A12 | 69.57±15.479 | 96.29±5.502 | -9.620 | ﹤0.001 |
| A13 | 65.17±19.774 | 93.43±7.089 | -7.958 | ﹤0.001 |
| A14 | 59.71±22.405 | 89.54±8.603 | -7.353 | ﹤0.001 |
| A15 | 51.83±21.745 | 88.03±9.871 | -8.968 | ﹤0.001 |
| A16 | 70.49±17.328 | 95.86±6.367 | -8.131 | ﹤0.001 |
| B1 | 58.74±17.404 | 90.94±10.474 | -9.378 | ﹤0.001 |
| B2 | 58.74±16.103 | 93.43±8.008 | -11.410 | ﹤0.001 |
| B3 | 58.09±15.367 | 91.20±10.740 | -10.450 | ﹤0.001 |
| B4 | 61.49±17.166 | 90.26±12.320 | -8.056 | ﹤0.001 |
| B5 | 63.31±16.694 | 89.17±13.838 | -7.055 | ﹤0.001 |
| B6 | 57.86±13.138 | 90.46±10.982 | -11.263 | ﹤0.001 |
| C1 | 66.14±14.011 | 94.37±7.220 | -10.596 | ﹤0.001 |
| C2 | 68.23±12.093 | 94.34±8.047 | -10.636 | ﹤0.001 |
| C3 | 72.14±12.825 | 95.89±5.640 | -10.026 | ﹤0.001 |
| C4 | 70.37±16.308 | 95.03±8.187 | -7.994 | ﹤0.001 |


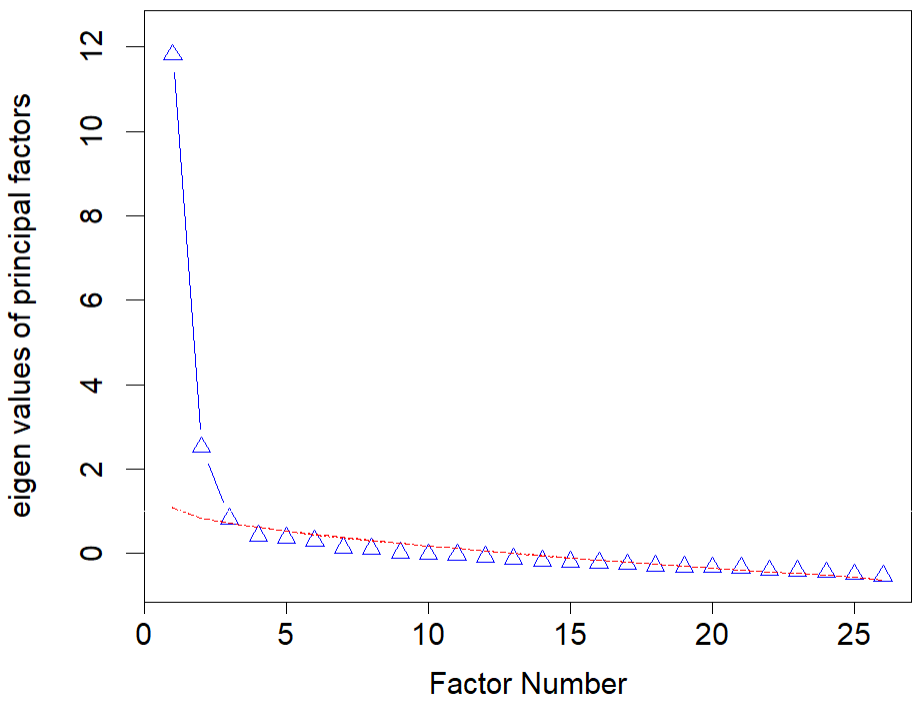


Figure S1 Scree plot

Table S2 Assumptions of unidimensionality of the 3 dimensions of the WOFI

| Dimension | KMO | Bartlett’s test | First eigenvalue | Second eigenvalue | Ratio | Unidimensionality |
| --- | --- | --- | --- | --- | --- | --- |
| PTF | 0.92 | *p*﹤0.001 | 3.78 | 0.02 | 189.00 | YES |
| NSF | 0.91 | *p*﹤0.001 | 3.87 | 0.06 | 64.50 | YES |
| DAF | 0.80 | *p*﹤0.001 | 1.83 | 0.11 | 16.64 | YES |


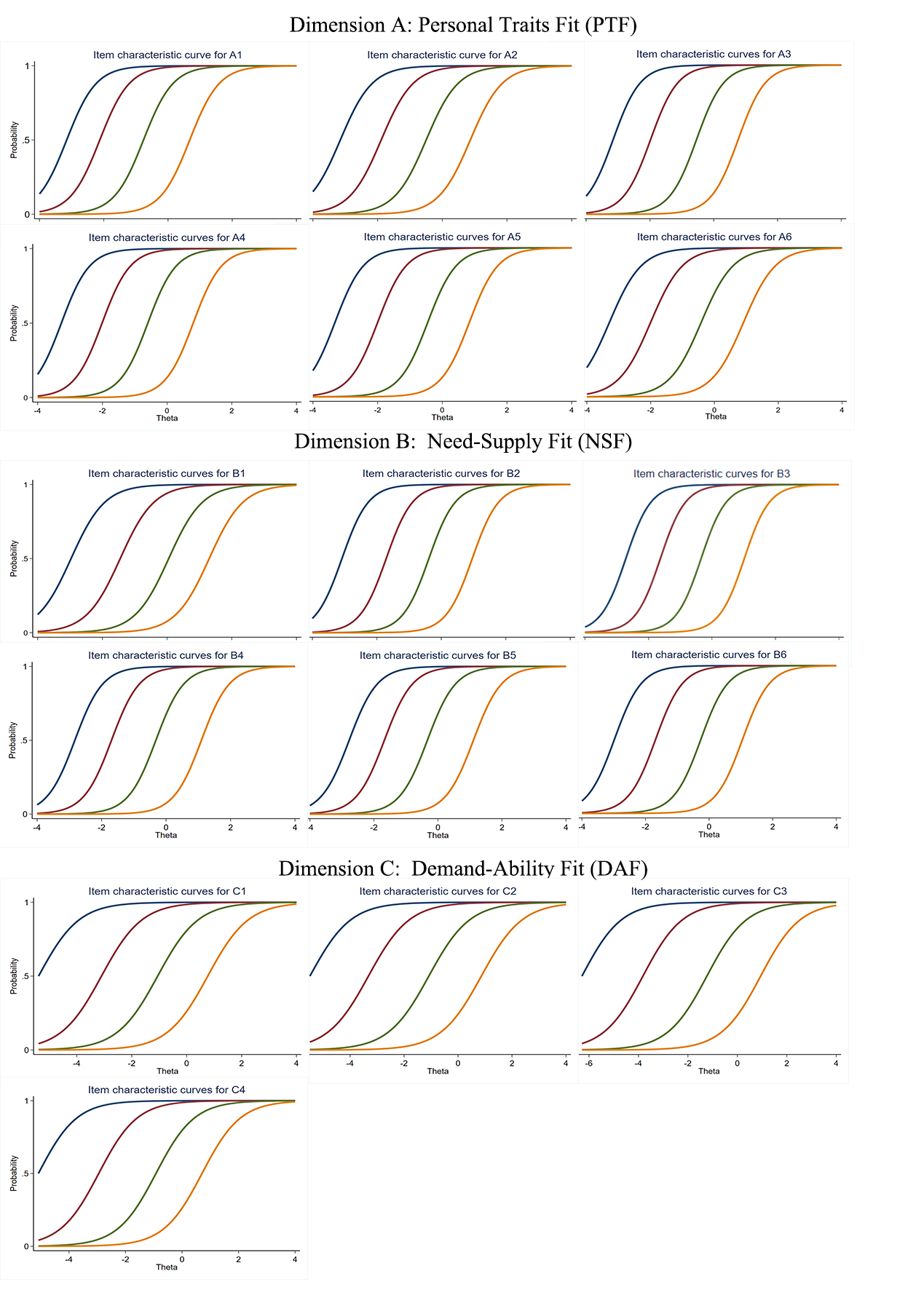


**Figure S2** Item characteristic curves for each item


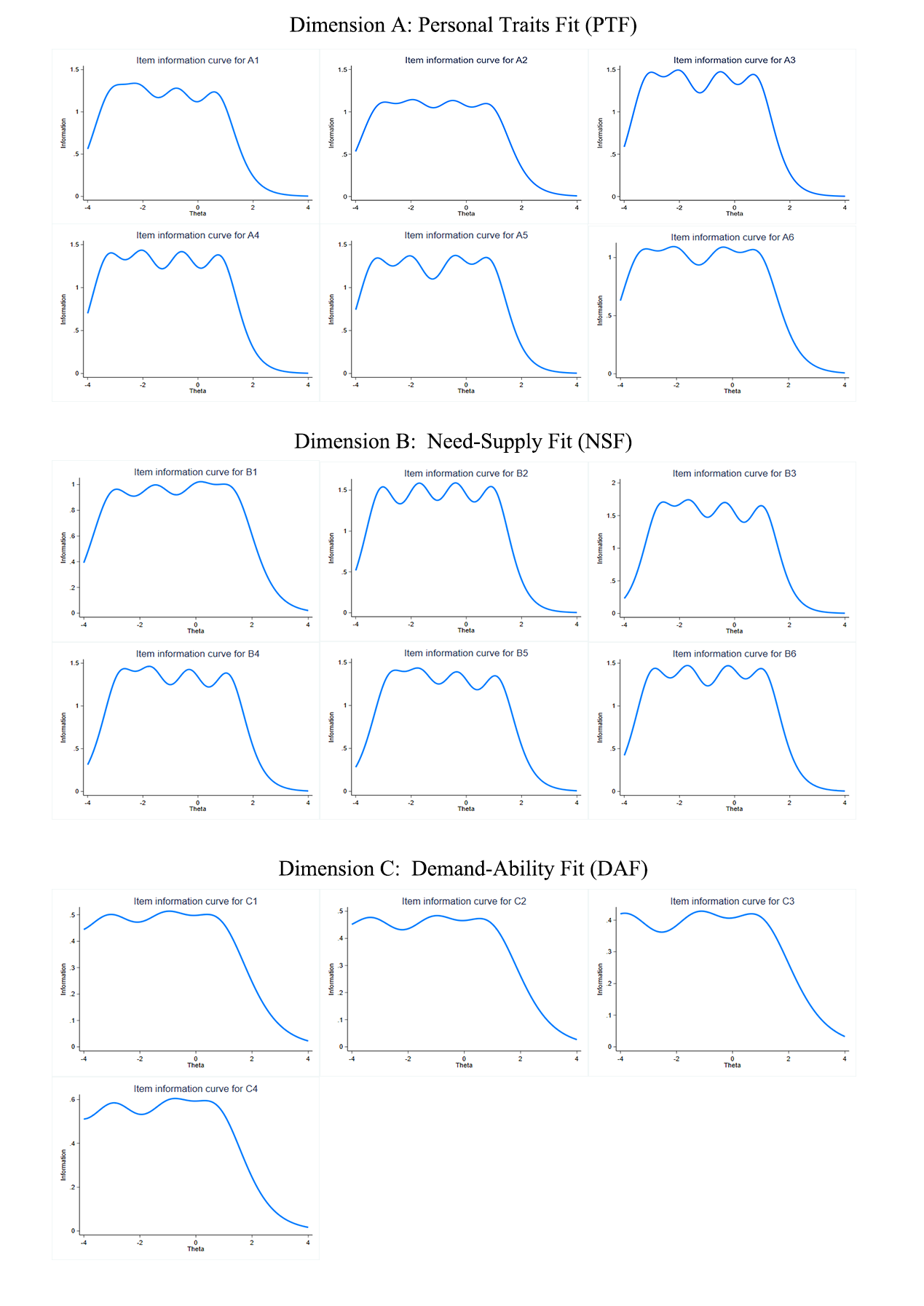


**Figure S3** Item information curves for each item
